# Zika Virus Envelope Protein Domain III Produced in *K. phaffii* Has the Potential for Diagnostic Applications

**DOI:** 10.3390/diagnostics12051198

**Published:** 2022-05-11

**Authors:** John Willians Oliveira Prates, Mariana Fonseca Xisto, Jojo Silva Rodrigues, João Pedro Cruz Colombari, Júlia Maria Alves Meira, Roberto Sousa Dias, Cynthia Canedo da Silva, e Sérgio Oliveira de Paula

**Affiliations:** 1 Department of Microbiology, Federal University of Viçosa, Viçosa 36570-900, Minas Gerais, Brazil; john_prates@hotmail.com (J.W.O.P.); jorodriguez6694@gmail.com (J.S.R.); ccanedo@ufv.br (C.C.d.S.); 2 Laboratory of Molecular Immunovirology, Department of General Biology, Federal University of Viçosa, Viçosa 36570-900, Minas Gerais, Brazil; marianaxisto@hotmail.com (M.F.X.); rosousa318@gmail.com (R.S.D.); 3 Department of Medicine and Nursing, Federal University of Viçosa, Viçosa 36570-900, Minas Gerais, Brazil; joao.colombari@ufv.br (J.P.C.C.); julia.meira@ufv.br (J.M.A.M.)

**Keywords:** Zika virus, protein E, domain III, *Komagataella phaffii*, diagnostic

## Abstract

Zika virus (ZIKV) represents a global human health threat and it is related to severe diseases such as congenital Zika syndrome (CZS) and Guillain-Barré syndrome (GBS). There is no vaccine available nor specific antiviral treatment, so developing sensitive, specific, and low-cost diagnostic tests is necessary. Thus, the objective of this work was to produce the Zika virus envelope protein domain III (ZIKV-EDIII) in *Komagataella phaffii* KM71H and evaluate its potential for diagnostic applications. After the *K. phaffii* had been transformed with the pPICZαA-ZIKV-EDIII vector, an SDS-PAGE and Western Blot were performed to characterize the recombinant protein and an ELISA to evaluate the antigenic potential. The results show that ZIKV-EDIII was produced in the expected size, with a good purity grade and yield of 2.58 mg/L. The receiver operating characteristic (ROC) curve showed 90% sensitivity and 87.5% specificity for IgM, and 93.33% sensitivity and 82.76% specificity for IgG. The ZIKV-EDIII protein was efficiently produced in *K. phaffi*, and it has the potential for diagnostic applications.

## 1. Introduction

Zika virus (ZIKV) is an arbovirus that belongs to the genus *Flavivirus* and the family *Flaviviridae* [1]. It was first identified in Uganda in 1947 in infected sentinel rhesus monkeys [2]. The ZIKV can be transmitted by *Aedes* spp. mosquitoes [3], as well as sexually [4], vertically [5], and by blood transfusion [6]. Most cases of infection are asymptomatic, while symptomatic cases may present with nonspecific symptoms such as fever, conjunctivitis, headache, rash, and muscle and joint pain [7]. In addition, ZIKV infection is also linked to severe diseases such as Congenital Zika Syndrome (CZS) [8] and Guillain-Barré Syndrome (GBS) in adults [9], and this led the World Health Organization (WHO) to declare ZIKV a Public Health Emergency of International Concern in 2016 [10]. However, no vaccine or specific antiviral exists against ZIKV [11]. Therefore, an accurate and cost-effective diagnosis is essential to identify new infections and control the disease.

ZIKV is an enveloped, positive-sense, single-stranded RNA genome virus of approximately 11 kilobases with a single ORF, which is translated into a polyprotein that is cleaved by viral and host proteases to give the structural (C, prM/M, and E) and the non-structural proteins of the viral particle (NS1, NS2A, NS2B, NS3, NS4A, NS4B, and NS5). The E protein is responsible for virus binding to the cell receptor, entry into the host cell, and membrane fusion [12]. Each E protein monomer has three domains: the central domain I (EDI), the dimerization domain containing a fusion peptide (EDII), and the receptor-binding domain (EDIII) [13,14]. The EDIII has a barrel structure that resembles an immunoglobulin domain formed by antiparallel β-sheets. This domain has sites for binding to the cell receptor and potent antigenic and immunogenic epitopes, stimulating the immune system to produce specific antibodies for ZIKV [15]. Those antibodies can be captured using an ELISA test containing recombinant EDIII as an antigen [16]. ZIKV anti-EDIII antibodies did not bind to Dengue virus type 2 (DENV-2) or West Nile virus (WNV) EDIII in the ELISA test, as observed by Sapparapu and colleagues (2016), indicating that these ZIKV anti-EDIII antibodies are specific for ZIKV [17]. Furthermore, the DENV anti-DIII polyclonal antibodies were also specific for DENV and did not induce ADE (antibody-dependent enhancement) for ZIKV, WNV, and YFV, meaning that there was no cross-reaction between these flaviviruses [18]. The use of EDIII is still more advantageous compared to the total envelope glycoprotein (E), which induces antibody formation with cross-reaction between flaviviruses, as reported by Campos and colleagues (2017) [18]. Therefore, Zika virus envelope protein domain III (ZIKV-EDIII) represents a great candidate antigen for diagnostic applications. 

The laboratory diagnosis of ZIKV infection depends on the stage of the disease and can be performed by a reverse transcription reaction, followed by real-time PCR (RT-qPCR), the Plaque Reduction Neutralization Test (PRNT), and by ELISA [19]. RT-qPCR is limited mainly by the short period of viral RNA detection in patients’ blood samples [20]. PRNT is a precise method, but it is time-consuming, complex, and not feasible for large-scale diagnosis [21]. In addition, it can be impaired by cross-reaction between flaviviruses [22]. The ELISA is widely and routinely used, and there are several commercially available kits for ZIKV, such as the Dia. Pro Zika virus IgM ELISA (Dia. Pro-Diagnostic Bioprobes, Sesto San Giovanni (MI), Italy) [23], and the InBios ZIKV Detect MAC-ELISA (InBios International, Inc., Seattle, WA, USA) [24]. However, due to genetic similarities, cross-reaction between flaviviruses can impair the performance of these tests and incorrect diagnosis can occur, especially in endemic regions where flaviviruses co-circulate [25,26,27]. Thus, there is a great need to develop new, rapid, sensitive, specific, scalable, and low-cost serological tests to aid in the diagnosis of ZIKV.

The choice of antigen production systems for developing new diagnostic tests is a crucial step and the yeast *Komagataella phaffii* (*Pichia pastoris*) is an advantageous alternative because it is recognized as an excellent system for the expression of heterologous proteins. The protein expression mechanism in this yeast is similar to that of mammalian cells, producing processed, active, and correctly folded recombinant proteins with high production yields. Furthermore, the genetics of *K. phaffii* is known, which facilitate the manipulation process. Finally, as the biotechnology industry desires, this expression system is fast, inexpensive, and has excellent potential for large-scale production [28,29].

Due to the advantages of *K. phaffii* as a heterologous expression system, this work’s objective was to produce ZIKV-EDIII in *K. phaffii* KM71H yeast and evaluate its potential use as an antigen for diagnostic applications. The recombinant yeast produced ZIKV-EDIII with a good yield and this protein showed high antigenicity in the ELISA. This work reinforces the efficiency of K. phaffii as an expression system and the potential of EDIII as an antigen for developing low-cost diagnostic tests for ZIKV. 

## 2. Materials and Methods

### 2.1. ZIKV-EDIII Gene and Constructing pPICZαA-ZIKV-EDIII

**Figure 1:**
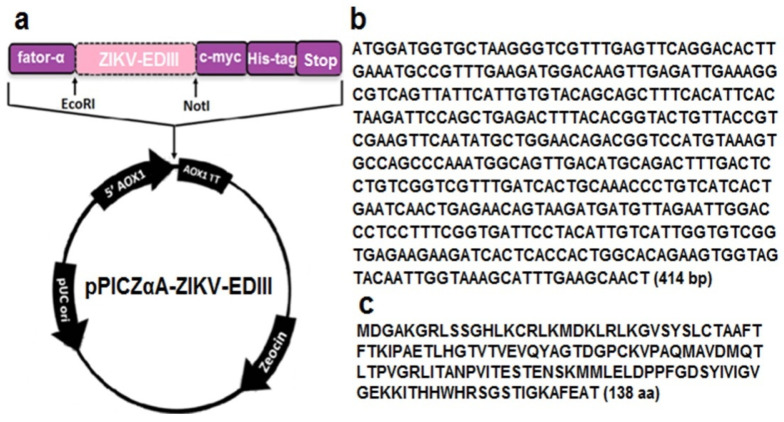
The pPICZαA-ZIKV-EDIII vector. (**a**) Schematic view of the expression cassette and the recombinant plasmid. (**b**) Genic sequence of ZIKV-EDIII optimized for expression in yeast. (**c**) Amino acid sequence of ZIKV-EDIII.

The ZIKV-EDIII gene sequence (414 bp, GenBank: MF352141.1) was codon-optimized for expression in yeast, synthesized and acquired in the pUC57 cloning vector (GenScript, Piscataway, NJ, USA), and flanked by EcoRI and NotI restriction enzyme sites (Promega, Fitchburg, MA, USA). Following the manufacturer’s recommendations, the plasmid pUC57 containing the ZIKV-EDIII gene was digested with the restriction enzymes EcoRI and NotI. The expression vector pPICZαA (Invitrogen, Carlsbad, CA, USA) was also digested with the same enzymes mentioned above. The ZIKV-EDIII insert released from pUC57 was cloned into pPICZαA using the T4 DNA ligase enzyme (Promega, Fitchburg, MA, USA), following the manufacturer’s recommendations. The resulting plasmid, named pPICZαA-ZIKV-EDIII (Figure 1a), contains the Shble gene for resistance to the Zeocin^TM^ antibiotic (Invitrogen, Carlsbad, CA, USA) and the AOX1 promoter, which controls ZIKV-EDIII gene expression. The expression cassette contains the following sequences: a factor-α signal sequence for secretory expression of the protein; ZIKV-EDIII genic sequence (Figure 1b); c-myc sequence for protein detection; and the C-terminal polyhistidine tail (6×His Tag) sequence, used in the detection and purification of the recombinant protein. The pPICZαA-ZIKV-EDIII was subjected to the PCR reaction with primers specific for ZIKV-EDIII (Foward:5’ gattgaaaggcgtcgtcagttattcat 3’ and Reverse: 5’ gacaccaatgacaatgtaggaatc3’). The amino acid sequence is presented in Figure 1c.

### 2.2. Generation of Recombinant K. phaffii

The plasmid pPICZαA-ZIKVEDIII was linearized with a SacI restriction enzyme (Promega, Fitchburg, MA, USA) and used to transform *K. phaffii* KM71H (Thermo Fisher Scientific, Waltham, MA, USA) by electroporation, as described by the manufacturer [30]. The selection of the transformants was performed in a YPDS medium (1% yeast extract, 2% peptone, 2% glucose, 1 M sorbitol, and 2% agar) containing the antibiotic Zeocin^TM^ at a concentration of 100 µg/mL. Genomic DNA from the transformants was extracted [31], and the presence of the plasmid of interest was verified by PCR with primers for the AOX1 region of pPICZαA-ZIKVEDIII (forward: 5’gacttggttccaattgacaagc3’and reverse: 5’gcaaatggcattctgacatcc3’) provided by GenOne (Genome Biotechnologies, Rio de Janeiro, RJ, Brazil). Genomic DNA from untransformed *K.phaffii* KM71H yeast was used as a negative control and the purified plasmid as a positive control.

### 2.3. Expression of ZIKV-EDIII

A positive clone was selected to produce ZIKV-EDIII, as described by [32]. The yeast was pre-inoculated in 16 mL of yeast extract, Peptone–Dextrose (YPD) medium (1% yeast extract, 2% peptone, and 2% dextrose), and incubated for 17 h at 30 °C under 270 rpm shaking. This pre-culture was inoculated in 500 mL of BMG (1.34% YNB, 0.002% biotin, 1% glycerol, 100 mM potassium phosphate—pH 6.0), and incubated at 30 °C, 270 rpm, until reaching OD_600_ = 10 (about 96 h). The cells were centrifuged at 3000× *g* for 10 min, washed with sterile distilled water, and solubilized in 80 mL of BMM medium (1.34% YNB, 0.002% biotin, 100 mM potassium phosphate—pH 6.0, 1% casamino acid, and 2% methanol) for protein expression. The culture was incubated at 20 °C, 270 rpm for six days. Methanol was added daily to the final concentration of 2%. After this period, the culture was centrifuged and the supernatant was collected for protein purification.

### 2.4. Purification of ZIKV-EDIII

The protein purification was performed by affinity chromatography using a 5 mL HisTrap^®^ Fast Flow Crude prepacked column (Cytiva, Marlborough, MA, USA) coupled to the AKTA purification system (Cytiva, Marlborough, MA, USA). The column was equilibrated with a binding buffer (20 mM sodium phosphate, 500 Mm NaCl, 20 mM imidazole, pH 7.4), and the same binding buffer was mixed with an equal volume of the supernatant collected from the culture, injected into the equipment, and analyzed at a flow rate of 5 mL/min. The column-bound protein fraction was recovered after applying an elution buffer (20 mM sodium phosphate, 500 mM NaCl, 300 mM imidazole, pH 7.4). The purified protein was concentrated by ultrafiltration using an Amicon^®^ Ultra Centrifugal Filter (Merck Millipore, Darmstadt, HE, Germany) and quantified by densitometry using ImageJ^®^ software (https://imagej.nih.gov/ij/ accessed on 6 June 2021). For this, a standard curve of BSA was performed in the following concentrations: 250 µg/mL, 125 µg/mL, 62.5 µg/mL, 31.25 µg/mL, and 15.625 µg/mL. The densitometry values of each protein were used to construct an analytical curve, obtaining its equation, which was used to estimate the concentration of purified ZIKV-EDIII.

### 2.5. Serum Samples

In this study, 121 human serum samples provided by the Oswaldo Cruz Foundation in Pernambuco, Brazil (FIOCRUZ/PE) were used. The patients’ blood samples were incubated for coagulation at room temperature for 30 min. Then, they were centrifuged at 3000 rpm for 20 min, and the sera were collected, inactivated at 55 °C for 30 min, and stored at −20 °C. Serum samples were confirmed positive/negative for ZIKV IgM/IgG using the Zika virus IgM/IgG ELISA kits (Euroimmun, Lübeck, SH, Germany) and theInBios ZIKV Detect MAC-ELISA (InBios International Inc., Seattle, WA, USA). All serums were tested according to the protocols approved by the Institutional Committee for Human/Animal Care and Use and the Ethics Committee. Serum samples were previously confirmed positive/negative for ZIKV IgM and IgG using ELISA. For IgM, 30 samples were positive, and 32 were negative, while for IgG, 30 were positive and 29 were negative. All samples were kept anonymous.

### 2.6. Protein Analyzes

The purified ZIKV-EDIII protein was electrophorized using Tricine SDS-PAGE 12% [33] and stained with Coomassie Blue (Invitrogen, Carlsbad, CA, USA). The protein was transferred to a nitrocellulose membrane for the Western Blot assay and blocked for 30 min with 3% gelatin in TBS-T buffer (NaCl 0.8%, KCl 0.02%, Tris 0.3%, 0.05% Tween 20). The membrane was then washed five times with TBS-T and incubated overnight with a pool of four human sera samples positive for ZIKV IgM. The sera dilution used was 1:1000. The membrane was washed five times with TBS-T and incubated with secondary antibody anti-human IgM conjugated with alkaline phosphatase (Sigma, St. Louis, MO, USA) for two hours under gentle agitation at room temperature. The membrane was rewashed with TBS-T and revealed with BCIP/NBT substrate (Sigma).

### 2.7. ELISA

ZIKV-EDIII (Figure 1c) was used as the coating antigen to sensitize two polystyrene 96-well microplates (Corning^®^, Tewksbury, MA, USA). For this purpose, the protein was diluted in a carbonate–bicarbonate buffer (50 mM, pH 9.6), transferred to the microplates (2 µg/well), and incubated at 4 °C overnight. As a control for the plates, the carbonate–bicarbonate buffer was added in triplicate as a reaction blank. The microplates were blocked with 3% gelatin in PBS buffer (10 mM, pH 7.2) and incubated for 30 min at 37 °C. Human sera positive/negative for ZIKV IgM and IgG were diluted 1/100 in PBS buffer with 1% gelatin and added in triplicate to microplates, followed by incubation at 37 °C for 3 h. Thirty positive and 32 negative serum samples for the IgM ELISA were used. The microplates were washed 5× with PBS containing 0.05% Tween-20 (PBS-T). Then, anti-human IgM and IgG antibodies (Sigma) at dilutions of 1/10,000 and 1/60,000, respectively, were added to the appropriate microplates, and the substrate 2,2’-Azino-bis (3-ethylbenzothiazoline-6-sulfonic acid (Sigma) was added, with incubation at room temperature for approximately 15 min. The reaction was stopped with 1% SDS, and optical density (OD) was evaluated in a Multiskan GO spectrophotometer (Thermo Scientific, Waltham, MA, USA) at a wavelength of 405 nm. This experiment was repeated on a different day as a biological replicate, and the mean absorbances of the plates were used for statistical analyses.

### 2.8. Statistical Analysis

The statistical analysis was performed using GraphPad Prism7 software (GraphPad Software, San Diego, CA, USA). The ROC curve was used to estimate the ELISA assay’s cut-off, sensitivity, and specificity. The unpaired *t*-test was performed to assess the significance of the assay.

## 3. Results

### 3.1. Generation of pPICZαA-ZIKV-EDIII Vector and K. phaffii Transformation

**Figure 2:**
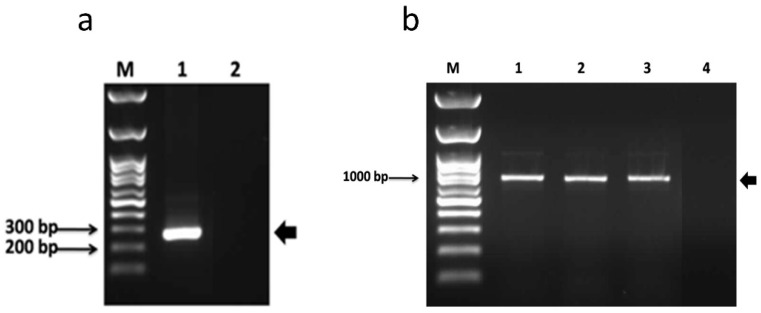
PCR confirmation of the pPICZαA-ZIKV-EDIII construct and transformation of *K. phaffii*. (**a**) PCR of pPICZαA-ZIKV-EDIII confirming the correct cloning of the cassette on the vector. M: molecular weight marker; 1: amplified fragment of pPICZαA-ZIKV-EDIII; 2: empty pPICZαA used as the negative control. (**b**) PCR of *K. phaffii* genomic DNA. M: molecular weight marker; 1–3: transformed *K. phaffii* clones; 4: untransformed *K. phaffii* (negative control).

The PCR of pPICZαA-ZIKV-EDIII confirmed the cassette cloning as a band size of about 270 bp, and the empty vector was used as a negative control (Figure 2a).

To verify the integration of the ZIKV-EDIII gene into the yeast genome, a PCR was performed with specific primers for the AOX1 promoter and EDIII. The cloning efficiency was confirmed in three yeast colonies, with an amplicon of approximately 1000 bp, consistent with the expected size of the target sequence of the primers used (total 1002 bp: AOX- 588 bp + ZIKV-EDIII- 414 bp). The DNA from the untransformed yeast was used as a negative control (Figure 2b). In the negative control, there was no amplification.

### 3.2. Expression, Purification, and Characterization of ZIKV-EDIII

After an initial screening, the best clone (protein expression) of transformed *K. phaffii* was grown in a BMM medium to induce recombinant protein expression. The protein was purified by affinity chromatography and concentrated with an Amicon^®^ Ultra Centrifugal Filter. A 10 mL sample volume was recovered after the concentration and quantified at 129 µg/mL using ImageJ^®^ software. The final yield obtained was 2.58 mg of ZIKV-EDIII protein in 1 L of culture (Table 1).

**Figure 3:**
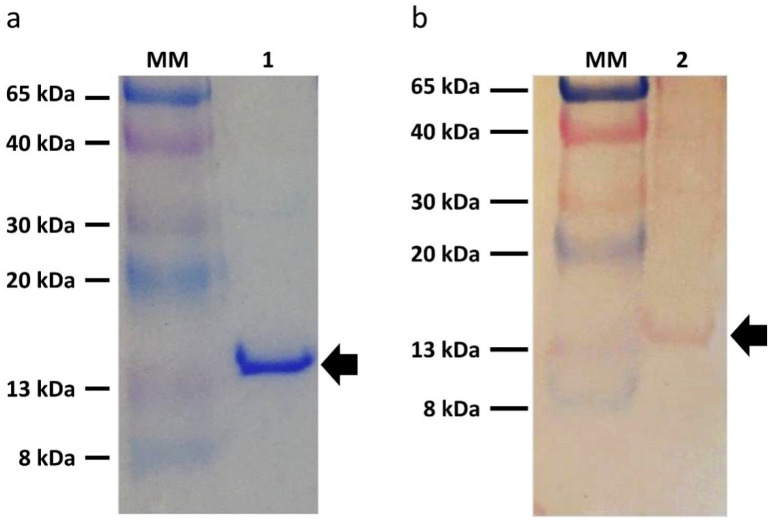
Characterization of ZIKV-EDIII. (**a**) SDS-PAGE 1: ZIKV-EDIII. (**b**) Western Blot. 2: ZIKV-EDIII. Arrows indicate recombinant protein.MM: molecular weight marker (Color burst^TM^ Electrophoresis Marker—Sigma).

To characterize ZIKV-EDIII, an SDS-PAGE and a Western blot were performed. The recombinant protein was detected as a 14 kDa band (Figure 3a), and confirmed by Western blot using polyclonal antibodies (Figure 3b).

### 3.3. IgM and IgG ELISA

**Figure 4:**
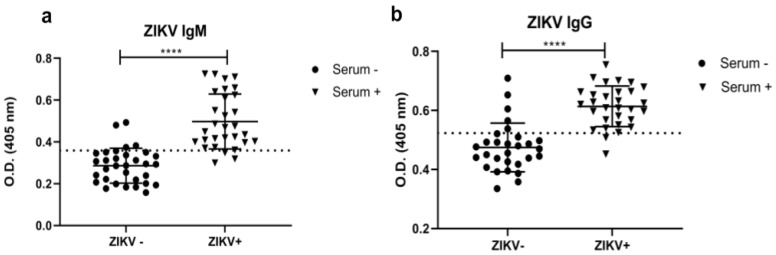
Anti-ZIKV indirect ELISA. (**a**) Anti-IgM and (**b**) Anti-IgG. ZIKV+ represents positive serum and ZIKV– represents negative serum. The dashed line shows the cut-off for each antibody isotype from the ROC curve, 0.3593 for IgM and 0.5234 for IgG. Each dot in the graph represents a different sample. **** refers to the *p* < 0.0001.

The ZIKV-EDIII was used as the antigen to detect anti-ZIKV antibodies (IgM and IgG) in the indirect ELISA assay. The plate controls had no absorbance reading and were used as a beacon for the wells containing recombinant protein. The IgM ELISA had a cut-off of 0.3593, with a 90% sensitivity and 87.5% specificity (Figure 4a, Table 2), while the IgG ELISA had a cut-off of 0.5234, with a 93.33% sensitivity and 82.76% specificity (Figure 4b, Table 2). The graphs were obtained with a 95% confidence interval from the ROC curve (Supplementary Figures S1 and S2, Supplementary Tables S1 and S2). Comparing the standard 3× SD of negative sera cut-off to the ROC cut-off was performed (Supplementary Table S3). The raw optic density values of the ELISA tests were provided in the supplementary material, with false positives and negatives highlighted (Supplementary Tables S4 and S5).

## 4. Discussion

ZIKV represents a threat to global human health and, with the global spread and possible association with severe disease, efforts have been made worldwide to develop vaccines and diagnostics [11,34]. Nevertheless, problems with sensitivity and specificity of serological tests have hindered the diagnosis of ZIKV, demanding the development of new methodologies to overcome these barriers [34]. In this context, aiming to obtain an antigen for the development of an accurate diagnostic test, we obtained promising results with the production of ZIKV-EDIII in *K. phaffii* and the evaluation of its antigenicity.

Due to the recognition of *K. phaffii* as an excellent expression system [29], this yeast was chosen in this work. In the first step, the gene encoding ZIKV-EDIII was optimized for expression in yeast, a strategy widely adopted to increase recombinant protein expression [35]. This gene was cloned under the control of the strong AOX1 promoter, which is very popular and induced with methanol [36]. Methanol is also used as a carbon and energy source by yeast due to two genes in its genome, AOX1 and AOX2, which encode enzymes that participate in methanol metabolism, allowing its utilization by yeast. Because of this methanol utilization ability, three phenotypes of *K. phaffii* exist: Mut^+^ (high methanol utilization), Mut^S^ (low methanol utilization), and Mut^−^ (no methanol utilization) [37,38]. Thinking about the large-scale production of recombinant proteins, the use of high amounts of methanol would not be adequate, considering that it is toxic and flammable, and new alternatives would be necessary. One of them would be the use of Mut^S^ yeasts, such as the KM71H used in this work, which needs low amounts of methanol to grow because they have only the functional AOX2 gene [39]. A second alternative would be to use a methanol substitute to induce the AOX1 promoter, such as formate, thus avoiding the problems associated with the use of methanol on a large scale [40].

The recombinant yeast produced ZIKV-EDIII in a soluble form and secreted this protein to the culture supernatant with a good yield (2.58 mg/L). This yield is higher than other works, which obtained 1.412 mg/L and 1 mg/L [41,42], and can be further maximized due to the possibility of using *K. phaffii* in the large-scale production of recombinant proteins [43,44]. The SDS-PAGE analysis showed the presence of a single band on the gel (Figure 3a), indicating good purity of the obtained protein. Thus, the low secretion of endogenous proteins can explain the good purity by *K.phaffii*, which makes the culture supernatant contain the recombinant protein of interest [45]. In addition, the culture medium used for expression of the recombinant protein was a minimal medium (BMM medium), which contains neither yeast extract nor peptone, i.e., the presence of protein is minimal. 

Although ZIKV-EDIII has been produced in different expression systems, such as bacteria, mammalian cells, insect cells, plants, and yeast, some points need improvement. Expression in *E. coli* has generated protein being retained in inclusion bodies, which need to be solubilized and refolded to recover the native conformation [46,47,48]. The 293T and *Drosophila* S2 cells produced ZIKV-EDIII efficiently and with adequate folding to present its native conformation [49,50]. However, compared to bacteria or yeast, these systems are traditionally expensive due to the high cost of culture media, supplements, transfection reagents, and other components needed to culture and maintain these cells [51,52]. The expression in the plant has also been reported as an up-and-coming system for large-scale production with low cost [53]. However, there is commercial resistance to its use, mainly due to the difficulties of further processing the plant tissue to recover the protein, considering that it is retained intracellularly, making this means of production more complex compared to yeast, for example [54]. The production in yeast has achieved good results, expressing ZIKV-EDIII with high quality, good yield, and in the native conformation [55], which proves it to be an efficient expression system for the production of this recombinant protein.

Western blot using polyclonal human ZIKV+ antibodies to detect ZIKV-EDIII confirmed the identity of this recombinant protein, as shown in Figure 3b. Accordingly, *K. phaffii* acted as an efficient expression system to produce arboviral proteins, which had already been demonstrated by Zhang and colleagues (2019) and Xisto and colleagues (2020) [55,56]. They expressed the EDIII ZIKV recombinant protein in PichiaPink^TM^ (Life Technologies, Carlsbad, CA, USA, EUA) yeast as a possible vaccine candidate and NS1 in *K. phaffii* for diagnostics purposes, respectively. Zhang and colleagues (2019) obtained a final yield of 4.5 mg/L, higher than this work, and the protein showed an ideal conformation, able to inhibit ZIKV infection in vitro. Despite the similarities with our work, the use of PichiaPink^TM^ may have conferred additional advantages regarding the yield obtained. According to the manufacturer, this yeast can produce a greater and more significant amount of protein, reaching the production of 12 g/L at scales above 1000 L [57,58].

The ZIKV-EDIII obtained in this work showed good sensitivity and specificity in the ELISA, estimated by the ROC curve analysis (Figure 4, Table 2). This result is comparable to those obtained by some commercially available kits. The CDC Zika MAC-ELISA showed a sensitivity of 90.9% for convalescent-phase samples, while the ZIKV Detect™ 2.0 IgM Capture ELISA showed 92.5% [59,60]. In contrast, Matheus and colleagues (2019) showed that Euroimmun Zika virus IgM ELISA showed a sensitivity of 49% and the Dia. Pro Zika virus IgM ELISA showed a sensitivity of 69% [61]. This low sensitivity reflects the difficulty of performing the diagnosis in endemic areas for flaviviruses. Similar to our work, Denis and colleagues (2019) evaluated the performance of a ZIKV-EDIII-based ELISA [16]. They obtained a good sensitivity and specificity for IgG at 92% and 90%, respectively, reinforcing the potential of this antigen for diagnostic applications.

Overall, our results show the ability of *K. phaffii* to produce ZIKV-EDIII with a good yield and purity. This recombinant protein was recognized by polyclonal human anti-ZIKV antibodies, indicating its antigenicity. 

## 5. Conclusions

These results show that ZIKV-EDIII was produced by *K. phaffii* is an antigen with the potential to be applied in serological diagnosis. However, although DIII is described in the literature with high specificity, more experiments need to be performed to evaluate the potential for a differential diagnosis with other arboviruses.

## Figures and Tables

**Table 1:** Quantification of ZIKV-EDIII by densitometry. Yield per liter of culture. Standard curve equation: y = 72.054x − 1249.5. R^2^ = 0.9925. The standard curve of BSA was constructed using the following concentrations: 250 µg/mL, 125 µg/mL, 62.5µg/mL, 31.25 µg/mL and 15.625 µg/mL.

	Densitometry	[ ] µg/mL	Volume (mL)	Yield (mg/L)
ZIKV-EDIII	8.070	129.3341	10	2.586682

**Table 2:** Sensitivity and specificity of the indirect ELISA. Unpaired *t*-test for significance. Receiver operating characteristic (ROC) curves were analyzed to estimate the diagnostic sensitivity and specificity.

	% Sensitivity	% Specificity	Cut-Off	*p*-Value
Anti-IgM	90.00	87.50	0.3593	<0.0001
Anti-IgG	93.33	82.76	0.5234	<0.0001

## Data Availability

The authors declare that all data supporting the findings of this study are available within the article.

## Supplementary Materials

The following supporting information can be downloaded at: https://www.mdpi.com/article/10.3390/diagnostics12051198/s1, Figure S1: ROC curve for anti-ZIKV IgM. The área under the curve was 0.9307 (95% confidence interval: 0.8685-0.9929) (*p* value: < 0.0001). The best cut-off was 0.3593, with 90 % sensitivity and 87.5 % specificity (blue dot); Figure S2: ROC curve for anti-ZIKV IgG. The area under the curve was 0.9017 (95% confidence interval: 0.8150-0.9885) (*p* value: < 0.0001). The best cut-off was 0.5234, with 93.33 % sensitivity and 82.76 % specificity (blue dot); Table S1: Sensitivity and specificity for anti-ZIKV IgM. The best cut-off is in line 31 and has been highlighted in bold; Table S2: of sensitivity and specificity for anti-ZIKV IgG. The best cut-off is in line 26 and has been highlighted in bold; Table S3: Comparison of ROC curve cut-off, sensitivity, and specificity with traditional 3xSD. The negative serum cut-off was obtained by averaging the optical densities of the ZIKV negative serum samples plus a three-fold standard deviation (Mean + 3xSD). The results were presented in the table below; Table S4: The optical density of the IgM ELISA was performed with ZIKV negative and positive human sera samples. The false positives are 17, 21, 18, and 13. The false negatives are: 7, 8 e 5. false-positive and false-negative samples are in bold; Table S5: The optical density of the IgG ELISA was performed with ZIKV negative and positive human serum samples. False positives are: 15, 10, 25,16 and 1. False negatives are 10 and 27. false-positive and negative samples are highlighted in bold.
