# Supplementary material for: Zika Virus Envelope Protein Domain III Produced in K. phaffii Has the Potential for Diagnostic Applications
Source: Diagnostics (Basel). 2022 May 11;12(5):1198. doi: 10.3390/diagnostics12051198 (PMC9139701; doi:10.3390/diagnostics12051198)
Supplement: Supplementary file 1 [file diagnostics-12-01198-s001.zip › diagnostics-1673335-supplementary.pdf]

SUPPLEMENTARY MATERIAL

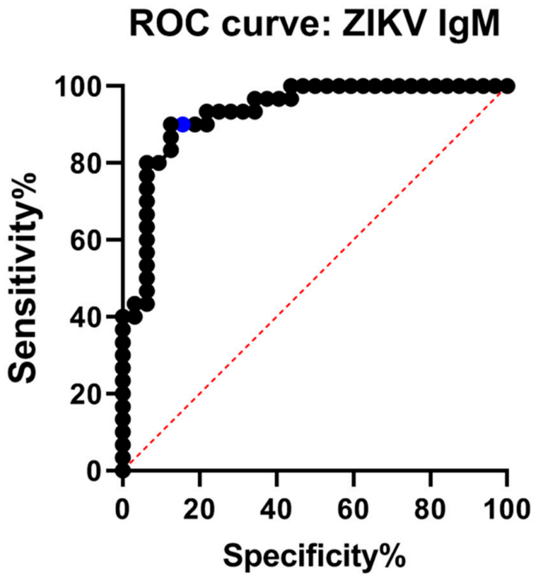

Supplementary Figure S1. ROC curve for anti-ZIKV IgM. The área under the curve was 0.9307 (95% confidence interval: 0.8685-0.9929) (P value: < 0.0001). The best cut-off was 0.3593, with 90 % sensitivity and 87.5 % specificity (blue dot).

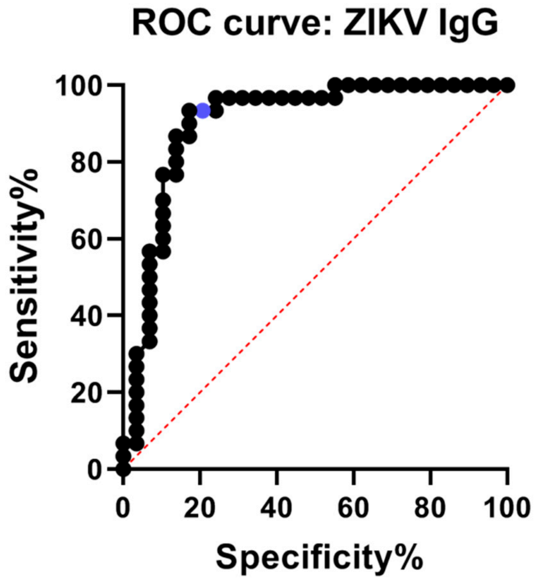

Supplementary Figure S2. ROC curve for anti-ZIKV IgG. The area under the curve was 0.9017 (95% confidence interval: 0.8150-0.9885) (P value: < 0.0001). The best cut-off was 0.5234, with 93.33 % sensitivity and 82.76 % specificity (blue dot).

Supplementary Table S1. Sensitivity and specificity for anti-ZIKV IgM. The best cut-off is in line 31 and has been highlighted in bold.

Line	Cut-off	Sensitivity%	Specificity%	Youden
1	> 0.1672	100	3.125	3.125
2	> 0.1801	100	6.25	6.25
3	> 0.1836	100	9.375	9.375
4	> 0.1888	100	12.5	12.5
5	> 0.1963	100	15.63	15.63
6	> 0.2000	100	18.75	18.75
7	> 0.2040	100	21.88	21.88
8	> 0.2130	100	25	25
9	> 0.2202	100	28.13	28.13
10	> 0.2302	100	31.25	31.25
11	> 0.2400	100	34,38	34.38
12	> 0.2473	100	37.5	37.5
13	> 0.2621	100	40.63	40.63
14	> 0.2782	100	43.75	43.75
15	> 0.2865	100	46.88	46.88
16	> 0.2901	100	50	50
17	> 0.2941	100	53.13	53.13
18	> 0.2976	100	56.25	56.25
19	> 0.3034	96.67	56.25	52.92
20	> 0.3093	96.67	59.38	56.05
21	> 0.3121	96.67	62.5	59.17
22	> 0.3158	96.67	65.63	62.3
23	> 0.3197	93.33	65.63	58.96
24	> 0.3259	93.33	68.75	62.08
25	> 0.3343	93.33	71.88	65.21
26	> 0.3414	93.33	75	68.33
27	> 0.3471	93.33	78.13	71.46
28	> 0.3496	90	78.13	68.13
29	> 0.3508	90	81.25	71.25
30	> 0.3540	90	84.38	74.38
**31**	> 0.3593	**90**	**87.5**	**77.5**
32	> 0.3665	86.67	87.5	74.17
33	> 0.3728	83.33	87.5	70.83
34	> 0.3782	80	90.63	70.63
35	> 0.3895	80	93.75	73.75
36	> 0.3987	76.67	93.75	70.42
37	> 0.4018	73.33	93.75	67.08
38	> 0.4064	70	93.75	63.75
39	> 0.4130	66.67	93.75	60.42

40	> 0.4181	63.33	93.75	57.08
41	> 0.4220	60	93.75	53.75
42	> 0.4297	56.67	93.75	50.42
43	> 0.4419	53.33	93.75	47.08
44	> 0.4591	50	93.75	43.75
45	> 0.4725	46.67	93.75	40.42
46	> 0.4779	43.33	93.75	37.08
47	> 0.4834	43.33	96.88	40.21
48	> 0.4897	40	96.88	36.88
49	> 0.5091	40	100	40
50	> 0.5339	36.67	100	36.67
51	> 0.5466	33.33	100	33.33
52	> 0.5804	30	100	30
53	> 0.6171	26.67	100	26.67
54	> 0.6322	23.33	100	23.33
55	> 0.6461	20	100	20
56	> 0.6555	16.67	100	16.67
57	> 0.6808	13.33	100	13.33
58	> 0.7069	10	100	10
59	> 0.7177	6.667	100	6.667
60	> 0.7248	3.333	100	3.333

Supplementary Table S2. of sensitivity and specificity for anti-ZIKV IgG. The best cut-off is in line 26 and has been highlighted in bold.

Line	Cut-off	Sensitivity%	Specificity%	Youden
1	> 0.3468	100	3.448	3.448
2	> 0.3725	100	6.897	6.897
3	> 0.3895	100	10.34	10.34
4	> 0.3937	100	13.79	13.79
5	> 0.4013	100	17.24	17.24
6	> 0.4126	100	20.69	20.69
7	> 0.4221	100	24.14	24.14
8	> 0.4321	100	27.59	27.59
9	> 0.4382	100	31.03	31.03
10	> 0.4398	100	34.48	34.48
11	> 0.4430	100	37.93	37.93
12	> 0.4477	100	41.38	41.38
13	> 0.4512	100	44.83	44.83
14	> 0.4539	96.67	44.83	41.5
15	> 0.4628	96.67	48.28	44.95
16	> 0.4734	96.67	51.72	48.39
17	> 0.4787	96.67	55.17	51.84
18	> 0.4836	96.67	58.62	55.29
19	> 0.4869	96.67	62.07	58.74
20	> 0.4898	96.67	65.52	62.19
21	> 0.4928	96.67	68.97	65.64
22	> 0.4954	96.67	72.41	69.08
23	> 0.5030	96.67	75.86	72.53
24	> 0.5092	93.33	75.86	69.19
25	> 0.5158	93.33	79.31	72.64
**26**	> 0.5234	**93.33**	**82.76**	**76.09**
27	> 0.5298	90	82.76	72.76
28	> 0.5360	86.67	82.76	69.43
29	> 0.5388	86.67	86.21	72.88
30	> 0.5415	83.33	86.21	69.54
31	> 0.5473	80	86.21	66.21
32	> 0.5583	76.67	86.21	62.88
33	> 0.5668	76.67	89.66	66.33
34	> 0.5749	70	89.66	59.66
35	> 0.5893	66.67	89.66	56.33
36	> 0.5972	63.33	89.66	52.99
37	> 0.6008	60	89.66	49.66

38	> 0.6047	56.67	89.66	46.33
39	> 0.6064	56.67	93.1	49.77
40	> 0.6084	53.33	93.1	46.43
41	> 0.6150	50	93.1	43.1
42	> 0.6229	46.67	93.1	39.77
43	> 0.6266	43.33	93.1	36.43
44	> 0.6303	40	93.1	33.1
45	> 0.6393	36.67	93.1	29.77
46	> 0.6495	33.33	93.1	26.43
47	> 0.6573	30	96.55	26.55
48	> 0.6623	26.67	96.55	23.22
49	> 0.6636	23.33	96.55	19.88
50	> 0.6718	20	96.55	16.55
51	> 0.6873	16.67	96.55	13.22
52	> 0.6960	13.33	96.55	9.88
53	> 0.6989	10	96.55	6.55
54	> 0.7052	6.667	96.55	3.217
55	> 0.7102	6.667	100	6.667
56	> 0.7330	3.333	100	3.333

Supplementary Table S3. Comparison of ROC curve cut-off, sensitivity, and specificity with traditional 3xSD. The negative serum cut-off was obtained by averaging the optical densities of the ZIKV negative serum samples plus a three-fold standard deviation (Mean + 3xSD). The results were presented in the table below:

ELISA	Mean + 3xSD	Curve ROC
Anti-IgM (cut-off)	0.33	0.3593
Anti-IgM (sensitivity)	93.33 %	90.00 %
Anti-IgM (specificity))	68.75	87.5 %
Anti-IgG (cut-off)	0.52	0.5234
Anti-IgG (sensitivity)	93.33 %	93.33 %
Anti-IgG (specificity)	79.3 %	82.76 %

Supplementary Table S4. The optical density of the IgM ELISA was performed with ZIKV negative and positive human sera samples. The false positives are 17, 21, 18, and 13. The false negatives are: 7, 8 e 5. false-positive and false-negative samples are in bold.

Human serum	ZIKV- (OD)	Human serum	ZIKV + (OD)
1	0.2069	1	0.4164
2	0.1937	2	0.4096
3	0.307	3	0.7242
4	0.241	4	0.5255
5	0.3456	**5**	**0.3485**
6	0.2872	6	0.3742
7	0.2706	**7**	**0.2998**
8	0.3203	**8**	**0.319**
9	0.1767	9	0.3968
10	0.1834	10	0.3713
11	0.1989	11	0.551
12	0.3569	12	0.3616
**13**	**0.3822**	13	0.4005
14	0.2011	14	0.435
15	0.3371	15	0.4243
16	0.3126	16	0.4755
**17**	**0.4802**	17	0.6589
**18**	**0.3742**	18	0.4031
19	0.2536	19	0.4488
20	0.2389	20	0.4197
**21**	**0.4927**	21	0.7254
22	0.3115	22	0.7111
23	0.3315	23	0.6401
24	0.351	24	0.6098
25	0.3506	25	0.7026
26	0.2858	26	0.6243
27	0.2929	27	0.4866
28	0,2214	28	0.6521
29	0.2953	29	0.5422
30	0.219	30	0.4694
31	0.1838	31	
32	0.1577	32	

Supplementary Table S5. The optical density of the IgG ELISA was performed with ZIKV negative and positive human serum samples. False positives are: 15, 10, 25,16 and 1. False negatives are 10 and 27. false-positive and negative samples are highlighted in bold.

Human serum	ZIKV- (OD)	Human serum	ZIKV + (OD)
**1**	**0.5379**	1	0.7548
2	0.3952	2	0.6618
3	0.4451	3	0.6042
4	0.3868	4	0.6077
5	0.3355	5	0.6791
6	0.4178	6	0.6627
7	0.3581	7	0.6965
8	0.3922	8	0.6209
9	0.487	9	0.7012
**10**	**0.6527**	**10**	**0.4521**
11	0.5213	11	0.5683
12	0.4804	12	0.5973
13	0.4502	13	0.5433
14	0.4978	14	0.5683
**15**	**0.7091**	15	0.7112
**16**	**0.5653**	16	0.597
17	0.4557	17	0.5815
18	0.4868	18	0.6527
19	0.4386	19	0.6462
20	0.4074	20	0.6324
21	0.5102	21	0.6282
22	0.4769	22	0.6249
23	0.4409	23	0.5255
24	0.4378	24	0.534
**25**	**0.6051**	25	0.609
26	0.4698	26	0.6644
27	0.493	**27**	**0.5081**
28	0.4264	28	0.6955
29	0.4925	29	0.5396
30		30	0.5513
